# Determinants of fertility in Malawi: Does women autonomy dimension matter?

**DOI:** 10.1186/s12905-022-01926-4

**Published:** 2022-08-15

**Authors:** James Forty, Kannan Navaneetham, Gobopamang Letamo

**Affiliations:** Department of Population Studies, University of Botswana, Gaborone, Botswana

**Keywords:** Women’s autonomy, Household, Fertility, Poisson, Malawi

## Abstract

**Background:**

Power inequality within the household and sexual relationships is linked to poor reproductive health. Malawi Government through National Sexual and Reproductive Health and Rights policy is committed to women empowerment as well fertility reduction. However, there is limited evidence in Malawi regarding whether women’s autonomy in the household is an independent determinant of fertility. With this background, the aim of this study is to investigate whether women’s autonomy in the household is a determinant of fertility in a poor socioeconomic and cultural setting.

**Methods:**

This study used Malawi Demographic and Health Survey, 2015–2016. A multivariable Poisson regression model was used to investigate if women’s autonomy in the household in Malawi determines fertility. The outcome measure, children ever born, was used as a measure of fertility. Women’s autonomy was measured with two dimensions, such as women’s household related decision makings and women’s sexual autonomy. The individual recode and household recode were merged for the analysis. The final study sample was 15,952 women who were cohabiting or married at the time of the survey.

**Results:**

The level of autonomy among women in the household related decisions and sexual autonomy was 49.1% and 64.0% respectively. Controlling for covariates, the study found no significant association between women’s autonomy dimensions in the household and number of children ever born. On the other hand, living in urban area (IRR = 0.91, CI 0.88–0.93); having less than tertiary education thus, no education (IRR = 1.83, CI 1.67–1.99) or primary education (IRR = 1.55, CI 1.42–1.69) or secondary education (IRR = 1.23, CI 1.13–1.33); poor households (IRR = 1.05, CI 1.01–1.09), starting cohabiting at the age of 19 years or less (AIRR = 1.15, CI 1.13–1.18) and not using modern contraceptive methods (AIRR = 1.17, CI 1.15–1.19) were significantly associated with fertility.

**Conclusions and recommendations:**

Though women’s autonomy does not have independent effect on fertility, it may be interacting with other sociocultural norms prevailing in the society. The study recommends that the Government of Malawi should come up with economic hardship emancipation policy for poor households. The government should also come up with a girl-child secondary school completion policy. Furthermore, the government should accelerate the implementation, monitoring and evaluation of National Gender Policy to ensure the women empowerment/autonomy is having positive effect at all level including the household.

## Background

Worldwide, studies have shown that power inequality within the household and sexual relationships is linked to poor reproductive health for women [1–4]. It is well known that fertility is one of the most important women reproductive health outcomes. Thus, high fertility rates, which lead to high population growth, have been pinpointed to hinder development and perpetuate poverty in developing countries [5]. Studies on the relationship between women autonomy and population health proliferated following the 1994 Cairo International Conference on Population and Development, which declared that human advancement is inextricably linked to advances in gender equality and equity, to the empowerment of women via their ability to control decisions related to their reproductive health and to the elimination of violence against women [6–9]. This global priority was reiterated later in the Millennium Development Goal [10]. Recently, the Sustainable Development Goals have provided impetus for continued action to tackle inequalities and empower all women and girls [11]. To accomplish the global agenda, Malawi Government through National Sexual and Reproductive Health and Rights policy is committed to improve women empowerment as well fertility reduction [12]. Nevertheless, the level of fertility is still high and slow pace of decline in Malawi. Therefore, the focus of this study is to understand the nexus between dimensions of women’s autonomy and fertility. The study findings could be extrapolated to the appropriate and strategic policy intervention for both women empowerment and fertility reduction.

At the household level, Women’s autonomy has been identified as one of the key dimensions that could change the prevailing high fertility in sub-Saharan Africa [13, 14]. Studies have made attempts to conceptualize and measure women’s autonomy and its various dimensions, such as economic, political and social sphere [15]. From the review of literature, autonomy is generally defined as freedom from external control or influences [15]. In other words, autonomy is the ability to formulate one’s own strategic choices, to control resources, and to exercise interpersonal control [16–18]. However, it is argued that autonomy in one setting does not necessarily translate into autonomy in another setting or facets of women’s lives [15, 19]. For instance, a woman may have autonomy at workplace but not at the household. Even within the household, different dimensions of autonomy may have distinct nature and quantum of association with fertility. Since autonomy is a multidimensional, Upadhyay et al. (2014) developed and validated a multidimensional instrument that can measure dimensions of autonomy in the household in the context of the United States of America [20]. The construct validity was demonstrated by a mixed-effects model in which women with sexual autonomy (unlike women with autonomy in household decision-making) was inversely associated with reproductive autonomy with respect to contraceptive use, pregnancy and childbearing. Moreover, autonomy has three levels: personal (Change in a person), relational (change in the relationships and power relations within a surrounding network) and environmental (changes in broader context) [21]. However, in this study, the focus is on relational autonomy in the household in terms of decision-making between women and partners or other members at the household. Studies in other context found that women’s autonomy in the household decision-making is significantly associated with fertility [22]. For instance, women decision-making on big purchases, daily purchases, own health care, own mobility were found to be associated with low fertility [23–27]. Studies in other countries have also shown that women’s sexual autonomy, especially asking partners to use condoms or refuse sex for justifiable reasons were found to be associated with fertility [28–30]. Few studies on the determinants of fertility in Malawi have shown that many socio-economic factors are associated with fertility [31–33]. These studies have not included women’s autonomy as a determinant of fertility. Further, there are limited evidence on the nature and direction of association between women’s autonomy and fertility in the context of Malawi. Thus, this study would fill this gap. In other words, this study attempts to investigate whether women’s autonomy in the household determines fertility (children ever born). As such, the findings of the study would provide greater policy implications regarding women empowerment and fertility reduction in Malawi.

### Theoretical framework: modernization theory

Many theories and frameworks explaining fertility change have been propounded [34]. The major explanation of fertility changes or dynamics has its origins in demographic transition theory (DTT) first developed by Thompson in 1929 and Notestein in 1945 [35]. This theory attributes the fertility declining to changes linked to the characteristics of modernization. This study attempts to explain women’s autonomy in the household and fertility nexus through modernization related theories. Modernization refers to a model of a progressive transition from a 'pre-modern' or 'traditional' to a 'modern' society [36]. The theory looks at the internal factors of a country while assuming that with assistance, "traditional" countries can be brought to development in the same manner more developed countries have been [36]. Modernization is widely debated in terms of development perspective as emphasized that economic development is an impetus for cultural process of human development that gives rise to an emancipating worldview, reflected in self-expression values that emphasize human choice and autonomy [37]. Since modernization theory is multidimensional [38], hence, its association to women’s autonomy (irrespective of dimension) and fertility can be well elaborated through its characteristics as explained below.

Urbanization is associated with decreasing fertility. White, et al., put forth that the children in urban areas are less likely to contribute to family income compared to rural (agricultural) areas [39]. Further, housing (space) is expensive in urban areas and there is more likelihood to access modern birth control and health services in general leading to low fertility in urban areas. In Malawi, the TFR of women living in urban areas is reported to be 3.0 children per woman, compared to a TFR of 4.7 among women of rural areas [40]. Regarding women’s autonomy, there is erosion of traditional barriers to mobility and self-expression in urban areas thus, giving way to new ways of thinking including enhancement of women’s autonomy [41].

Education is not just one of many socio-economic factors that matter, it is the single most important source of empirically observable population heterogeneity [42]. Interaction between education and fertility rate is realized through rewards associated with formal employment for educated women, who then forgo childbearing [43]. Educated women aspire to having a better life for their child in the future thus, they spend more on the children’s education, and they tend to have fewer numbers of children [44, 45]. In Malawi, it is reported that the TFR for women with at least secondary education as 2.8 children, while TFR for women with less than secondary education is 5.2 children [40]. Education is also lauded as a critical enabling factor for women’s autonomy and central to development goals [46]. Further, women’s schooling enhances cognitive abilities which are essential to women’s capacity to participate, to reflect on and act on the conditions of their lives and gain access to knowledge, information and ideas that would help them to do so [46].

Regarding household’s economic status, the initial proposition of microeconomic theory of fertility put forth that a reduction in the cost of a child or increase in a household’s economic status which leads to an increase in fertility as the household can afford to have a greater number of children [47]. However, this was later refined to an assumption that the couples are expected to respond to an increase in their household economic status by investing more in each child hence, they are likely to have fewer number of children [48]. This proposition was supported by the argument that economic status is always highly correlated with modernized and educated people who tend to be rational about decision-making and believes that quality of children is valued than quantity [49]. In Malawi, women from low and middle-income households are reported to have a TFR of 5.2 while women from rich households are reported to have a TFR of 3.5 [40].

Studies have argued that there is incompatibility between employment and childbearing. This is because employment especially formal employment demands time hence, it is inflexible for child bearing and upbringing [50, 51]. Moreover, it is also found women who work in more collectivized environments (including formal occupations) have fewer children than women who work in more individualized places (more informal) and those who do not work outside the home [52]. It is also argued that women’s paid employment is an important determinant of their autonomy [20]. The idea underlying this approach is that women’s employment can lead to a radical transformation in their options for economic survival and their bargaining power within families, including the ability to advocate for their own fertility desires [53].

Modernization as a pattern of social change has influenced each aspect of life, including religion and ethnicity [53]. Unlike in preindustrial societies where strong family ties and powerful religious beliefs enforce conformity and discourage diversity and change; modern values which foster efficiency have little reverence for the past thus, modern people adopt whatever social patterns allow them to achieve their goals [54]. Thus, modernization promotes a more rational and scientific worldview as tradition loses its hold thus, people gain more and more individual choice [55]. In Malawi, religion and ethnicity are found to be associated with fertility [56].

Mass media is an important aspect of modernization, as Lerner (1958) argued that media messages would enable audiences to identify with people and ideas that are different and distant from them [57]. Regarding fertility and mass media association, theoretical models of diffusion, ideational fertility and social interaction hold that individuals, communities, and nation-states interact with each other, spreading information, ideas, and technology regarding contraception and fertility ideals [58, 59]. Diffused ideas and technologies are received and reinterpreted, gaining new meaning in different contexts and impelling or constraining actions pertaining to women autonomy as well as fertility choice [60].

Empirically, in Malawi, the aforementioned characteristics of modernization have been found to be associated with fertility (31–33). However, the association is argued to be mediated by what is called as ‘proximate determinants of fertility’ [61, 62]. This study has used contraception use and age at first cohabitation [63] as proximate determinants of fertility. In Malawi, a study shows that the increased contraceptive prevalence rate and increased age at first marriage were found to be associated with declining fertility from 1992 to 2010 [64].

Despite the theoretical explanations of the association between the characteristics of modernization and women’s autonomy in the household, there is limited evidence in Malawi. Likewise, despite dimensions of women’s autonomy in the household found to be associated with fertility in other countries including sub-Sahara African countries [23–30], there is limited evidence of such association in Malawi. It is against this background that this study has looked at the association between dimensions of women’s autonomy in the household and fertility specifically number of children ever born. Studies including in Malawi have stated that socio-economic variables under consideration in this study as characteristics of modernization are associated with fertility. Further, as alluded to, modernization characteristics are associated with women’s autonomy and therefore this study controlled those factors to examine the independent effect of women’s autonomy on fertility.

### Conceptual framework

**Fig. 1:**
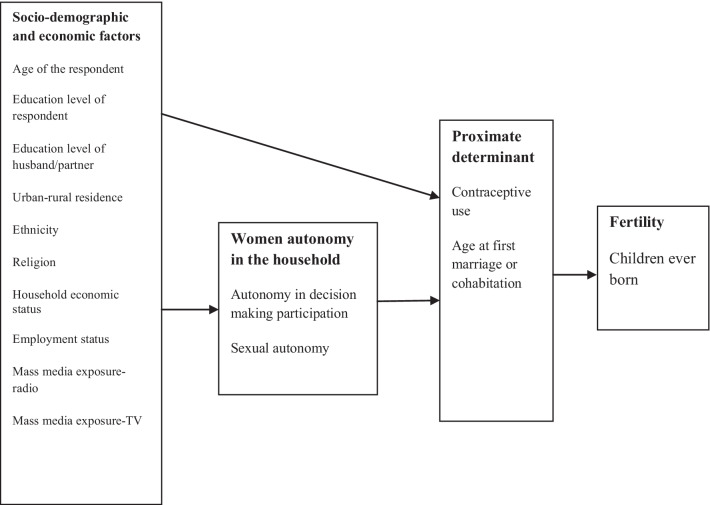
Conceptual framework of possible mechanisms through which women autonomy impacts upon fertility, adopted [37]

The conceptual framework (Fig. 1) shows that dimensions of women’s autonomy in the household as determinant of fertility but through the proximate determinants. The conceptual framework has been derived based on the review of literature including theoretical framework as discussed earlier. Several studies have shown that women’s household decision-making and sexual autonomy are associated with fertility [22, 26, 28, 65, 66]. This paper conceptualized that the socioeconomic characteristics of the women determine their household decision-making and sexual autonomy, and this in turn affect fertility through proximate determinants [67].

## Methods

### Study area

Located in Southern Africa, Malawi is landlocked and shares its borders with Mozambique, Zambia and Tanzania [68]. Malawi’s Population and Housing Census Report of 2018 puts the country’s population at 17,563,749, a 35% increase over the 2008 population of 13,029,498, increasing at an annual growth rate of 2.9% [39]. The report also shows that 47.2% of the total population is women of reproductive age (15–49 years), 47% of girls are already married by the age of 18 and 29 per cent of those aged 15–19 years have begun childbearing which contribute to 25% of all pregnancies annually. The total fertility rate in 2015/16 was 4.4 births per woman, down from 5.3 in 2010 and 6.7 in 1992 [41]. Poverty is high with the latest figures show the national poverty rate increased slightly from 50.7% in 2010 to 51.5% in 2016, but extreme national poverty decreased from 24.5% in 2010/11 to 20.1% in 2016/17 [68]. With most women participating in predominant low agriculture sector coupled with volatile economic growth, most of the women are not economically independent [69, 70]. This may exacerbate their lack of autonomy, which in turn inhibit them from making their own reproductive decisions.

However, Malawi is committed to ease the plight of the women as there is a National Gender Policy in place from 2000 and was revised in 2015. The policy aims to raise awareness of gender matters, legal rights of women, diet and the efficient utilization of food and nutrition, and the economic empowerment of women in conjunction with the poverty alleviation program [71]. Another important aspect of the National Gender Policy is better access to reproductive health services for women, which involves making family planning and other health facilities available to women in all parts of the country. Thus, a study on women autonomy and fertility nexus is relevant as it would inform the policy if it is having a significant impact on reproductive outcomes.

### Data source and sampling design

The study utilized secondary data from Malawi Demographic Health Survey (MDHS) for the year 2015/16. The individual recode and household recode files were merged for the analysis. The MDHS were stratified into urban and rural areas, and the multistage sampling design was used for selecting the respondents. At the first stage, 850 enumeration areas (EAs) were selected, of which 173 EAs were from urban areas and 677 EAs were from rural areas. At the second stage, 27,531 households were selected from which women were interviewed. Of the targeted 25,146 women, 24,562 women were successfully interviewed giving the response rate of 97.7% [40]. The target population of this study was cohabiting women. This generated a sub-sample of 15,952 women, a study population for the analysis in this paper, who were married or cohabiting at the time of survey.

### Measurement of variables

#### Dependent variable

The outcome variable is Children Ever Born (CEB). This is commonly used as a measure of lifetime fertility for understanding its determinants [72]. Children ever born is the number of children born alive to the woman in her lifetime fertility [72].

#### Independent variables

The main explanatory variables are women’s autonomy, measured in terms of household decision-making and sexual autonomy. Women’s autonomy of household decision-making variable was derived from three questions such as (a) person who usually decides on the health care of the respondent, (b) person who usually decides on large household purchases and (3) person who usually decides on visits to family or relatives. The responses such as respondent alone, respondent & partner and respondent and other person were recoded as participating in decision making coded as 1 (Yes). On the hand, the responses such as husband/partner alone, someone else and others were recoded as not participating in decision making which is coded as 0 (No). The composite index of women autonomy was derived as follows: (a) if women participated in all three-household decision-making coded as 2 (have autonomy); (b) if women participated in either one or two household decision making were coded as 1 (partial autonomy) and (c) if women did not participate in any household decision-making were coded as 0 (no autonomy). Similarly, sexual autonomy was derived based on responses from two questions, such as respondent can refuse sex (yes/no) and respondent can ask husband or partner to use condom (yes/no). The sexual autonomy index was derived as follows: (a) if women said yes to both questions were coded as 2 (have autonomy); (b) if women said yes to any one of the questions coded as 1 (partial autonomy) and (c) if women no to both questions were coded as 0 (no autonomy). Similar approach has been followed in other studies for measuring women’s household autonomy and sexual autonomy [72, 73].

The other independent variables used in this study are education level of the woman and her partner’s, urban–rural residence, ethnicity, religion, household income, woman’s occupation and age of a woman, age at first cohabitation and contraceptive use. These independent variables were selected for inclusion in the analysis based on the literatures reviewed that were significantly associated with fertility [74–92]. These variables have been categorized in a conventional way as used in other studies.

### Statistical analysis

To examine the factors influencing fertility, three statistical approaches were used. First, descriptive univariate analysis was performed to inspect the frequency distributions of the variables categories. Second, bivariate analysis was used to examine the relationships between the independent variables and number of children ever born. Since the dependent variable (CEB) is a continuous variable, the relationship between the mean number of children ever born, and the independent variables was analyzed using one-way ANOVA and F-test. Analysis of variance test was done to determine the fertility differentials. Lastly, the effect of the main explanatory variables and covariates on outcome variable was analyzed by using three Poisson regression models. Thus, model 1 measured unadjusted effect of each independent main variable (autonomy in decision-making and sexual autonomy in the household) on the number of children ever born, model 2 measured the net effect of each proximate determinant of fertility (modern contraceptive use and age at first cohabitation) and each independent main variable on the outcome variable and model 3 (included all control variables under consideration) measured the net effect of each variable on outcome variable.

### Analysis of variance (ANOVA)

In performing the ANOVA, the study took into consideration the assumptions, namely independence of the observations, normal distribution and homogeneity of variances as recommended by researchers [93, 94]. The assumption of independent observation was met, as DHS surveys ensured that in a sample a woman is interviewed once. The assumption of normal distribution was not met as Kolmogorov–Smirnov normality test that was done on outcome variable (number of children ever born) showed a significance of less than 0.5 thus, suggesting significant deviation from normal deviation. However, the sample size was large enough which researchers recommend that under such instance ANOVA can be used even if the normal distribution lacks [93]. The Test for homogeneity of variances assumption was done. Homogeneity of variance is an assumption underlying both t-tests and F-tests (ANOVA) in which the population variances (i.e., the distribution, or “spread,” of scores around the mean) of two or more samples are considered equal [95]. The assumption of homogeneity of variance was not met with all variables as such alternative F statistics (Welch’s or Brown–Forsythe) were used to determine if there was statistical significance as recommended by researchers [96, 97].

Furthermore, post-hoc Tests were done. Post hoc-tests are tests of the statistical significance of differences between group means calculated after having done ANOVA that shows an overall difference [61]. The reason for performing a post-hoc test is that the conclusions that are derived from the ANOVA test have limitations. It only provides information that the means of the three groups differ, and at least one group may show a difference. This means that it does not provide information on which group differs from which another group. As a result, there is a need for post-hoc tests to compare by pairing groups and verify which group differs from which another. Since the equal variance assumption was not met, the recommended post-hoc tests under such situation were done thus Temhane, Dunnett T3 and Games-Howel [93]. The tests produced almost similar results. Since ANOVA is limited to the association between one independent variable and the dependent variable, a multivariable poisson regression model was used to estimate the net effect of the women autonomy on fertility by controlling other covariates.

### Poisson regression model

Poisson’s distribution considers discrete/count outcome variables [98]. Poisson’s regression is more suitable for the count outcome variable, CEB.

The Poisson regression model takes this form [66],

$$\ln (\hat{\mu }) = {\text{ln}}\left( {{\text{time}}} \right) + {\text{b}}_{0} + b_{{1}} X_{{1}} + {\text{b}}_{{2}} {\text{X}}_{{2}} + \cdots + {\text{b}}_{{\text{p}}} {\text{X}}_{{\text{p}}} + {\text{ e}}$$

where $\hat{\mu }$ is the predicted count of the outcome variable given the specific values on the predictors *X*_1_, *X*_2_, …, *X*_*p*_. Where ln refers to the natural logarithm, *b*_0_ is the intercept, and *b*_1_ is the regression coefficient for the first predictor, *X*_1,_ etc. and ln(time) represent an offset variable as explained below. The use of Poisson error structure (e) resolves the problems with applying Ordinary Least Squares regression to count outcomes, namely, non-constant variance of the errors and non-normal conditional distribution of errors.

The coefficients were exponentiated to yield incident rate ratio (IRR) to ease interpretation of the results. The incident rate ratio explains how a change in X (independent variable) affects the rate at which the outcome variable occurs [99]. Thus, the results of the Poisson regression analysis have been presented and interpreted as IRR with 95% Wald confidence interval (WCI) [72]. Data analyses were performed using SPSS version 25 using appropriate survey weights.

The assumption of Poisson regression model, particularly on the equal variance, was tested using Akaike’s Information Criterion (AIC) and Bayesian Information Criterion (BIC). In the case of violation of equal variance assumption, negative binomial model was recommended [100]. There was a violation though under-dispersion (the conditional variance is less than predicted mean). However, much concern is related to over-dispersion, thus conditional mean is greater than predicted mean [66]. Notwithstanding, this study applied a Negative Binomial model to correct the under-dispersion. Nevertheless, it is also recommended that the best fit model between Poisson regression model and Negative Binomial model is the one with lower AIC and BIC [100]. Thus, both Poisson and Negative Binomial models were applied to the data sets. Poisson’s regression model produced lower AIC and BIC indices, and therefore this paper reported and interpreted results from it. Additionally, the Poisson regression model is applicable when counts have been made within a fixed interval of time (i.e., the measurement period has the same length for all cases). However, in situations where counts (e.g., number of children in a family) are made over varying periods of time across cases (e.g., age of the mother), then it becomes necessary to control for differences in the length of the periods in which observations are made. This has been accomplished through the incorporation of an offset variable, which takes a form of natural logarithm [98]. Thus, this study computed the variable, current age of women, as a natural log and then incorporated the newly computed variable as the predictor [99, 101, 102].

DHS sample is weighted and Malawi Demographic and Health Survey is no exception [40]. The weighting variables thus, hv005 for household and v005 for women were used in building complex sample analysis procedure in SPSS especially for descriptive analysis. However, there is still substantial discussion and controversy in the mathematical statistics on literature about the use of weights. Meanwhile, the consensus in the literature is that the weights should be used for descriptive statistics and there are fewer consensuses on whether the weights should be routinely used in multivariate models such as regression [103–106]. Hence, the application of complex sample analysis was limited to descriptive statistics.

## Results

### Characteristics of the respondents

The study population characteristics are presented in Table 1. The Table 1 revealed that about half of the respondents (49.5%) had autonomy, slightly above one-third of the respondents (36.3%) had partial autonomy and about one-seventh (14.2%) of the respondents had no autonomy in household decision making-participation. The second main independent variable, sexual autonomy, revealed that about two-third (64.1%) of the respondents had the autonomy, about one-fifth (18.6%) of the respondents had partial autonomy and about one-sixth (17.3%) of the respondents had no autonomy.

**Table 1:** Sample characteristics of married or cohabiting women aged 15–49 years at the time of survey, 2015–16 MDHS (N = 15,952)

Variable	N	%
*Autonomy in household decision making participation*		
Have autonomy	7896	49.5
Have partial autonomy	5791	36.3
Have no autonomy	2265	14.2
*Sexual Autonomy*		
Have autonomy	10,062	64.1
Have partial autonomy	2920	18.6
Have no autonomy	2715	17.3
*Age at first cohabitation*		
19 years and less	12,058	75.5
20 years and above	3894	24.5
*Contraceptive use*		
Using modern methods	9220	57.8
Not using modern methods	6732	42.2
*Religion*		
Christian	13,960	88.1
Muslim	1886	11.9
*Ethnicity*		
Chewa	4655	31.5
Tumbuka	1164	11.5
Lomwe	2774	18.0
Tonga	0580	3.8
Yao	1782	11.6
Sena	0812	5.3
Nkhonde	0204	1.3
Ngoni	1978	12.8
Mang’anja	0368	2.4
Nyanga	0288	1.9
*Type of place of residence*		
Urban	3031	19.0
Rural	12,921	81.0
*Highest education level*		
No education	2190	13.1
Primary	10,241	64.2
Secondary	3238	20.3
Higher	383	2.4
*Household economic status*		
Poor	5807	36.4
Middle income	6365	39.9
Rich	3780	23.7
*Husband/partner’s level of education*		
No education	1456	9.2
Primary	8377	53.2
Secondary	5008	31.8
Higher	915	5.8
*Employed*		
No	5328	33.4
Yes	10,624	66.6
*Exposure to media-radio listening*		
Not at all	7673	48.1
Less than once a week	2744	17.2
At least once a week	5535	34.7
*Exposure to media-watching television*		
Not at all	12,712	79.7
Less than once a week	1285	08.1
At least once a week	1955	12.3

With regard to proximate determinants of fertility under consideration; age at first cohabitation, the Table 1 indicated that about three-fourths (75.5%) of the respondents were married at the age of 19 years or less while about one-fourth (24.5%) of the respondents were married at the age of 20 years and above. More than half (57.8%) of the respondents used modern methods of contraception. The majority of the respondents (88.1%) were Christians and the proportion of the Muslim respondents was 11.9%. The majority of the respondents (81.0%) lived in rural areas. The higher percentage of the respondents (64.2%) had primary education, followed by respondents with secondary education (20.3%) while 13.1% of the respondents were not educated and women with higher education were the least (2.4%).

The Table 1 also revealed that majority of the respondents (39.9%) was from middle income households followed by respondents from poor household (36.4%) and respondents from rich households were the lowest (23.7%). Women who were not listening to radio at all were in majority (48.1%) followed by respondent who were listening to radio at least once a week (34.7%) while respondents who were listening to radio less than once a week were the least (17.2%). On the other hand, respondents who were not watching television at all were in majority (79.7%) followed by respondents who were watching television at least once a week (12.3%) while respondents who were watching television less than once a week were the least (8.1%).

## Association between women’s autonomy and fertility: bivariate analysis

The association between fertility and each of the independent variables was analyzed using one-way ANOVA, and the results are summarized in Table 2. Autonomy in household decision-making was not found to be significantly associated with number of children ever born (F = 0.923, P-value = 0.397). On the other hand, sexual autonomy had a significant association (F = 32.94, P-value = 0.000) with number of children ever born. The Post-hoc comparison tests revealed that the mean score for respondents with autonomy was significantly different from mean scores for respondents with partial autonomy and respondents with no autonomy. However, the mean scores for women with partial autonomy and women with no autonomy were not significantly different from each other.

**Table 2:** Bivariate analysis (based on ANOVA) of the association between women’s autonomy in the household including other covariates and fertility

Variable	Mean	95% CI	F-score	Significance
*Autonomy in household decision making participation*				
Have autonomy	3.47	3.42–3.52	0.923	0.397
Have partial autonomy	3.44	3.38–3.50		
Have no autonomy	3.41	3.31–3.50		
*Sexual Autonomy*				
Have autonomy	3.33	3.29–3.38	32.94	< 0.001
Have partial autonomy	3.57	3.48–3.65		
Have no autonomy	3.70	3.61–3.80		
*Age at first cohabitation/marriage*				
19 years and less	3.58	3.54–3.62	163.92	< 0.001
20 years and above	3.04	2.98–3.11		
*Modern Contraceptive use*				
Using modern methods	3.71	3.66–3.75	277.83	< 0.001
Not using modern methods	3.10	3.04–3.16		
*Religion*				
Christian	3.44	3.40–3.48	1.228	0.293
Muslim	3.52	3.41–3.64		
*Ethnicity*				
Chewa	3.51	3.44–3.58	1.75	0.072
Tumbuka	3.42	3.31–3.52		
Lomwe	3.39	3.31–3.48		
Tonga	3.28	3.11–3.46		
Yao	3.41	3.30–3.52		
Sena	3.51	3.36–3.67		
Nkhonde	3.35	3.04–3.66		
Ngoni	3.40	3.31–3.50		
Mang’anja	3.72	3.50–3.94		
Nyanga	3.43	3.18–3.67		
*Type of place of residence*				
Urban	2.83	2.77–2.90	275.86	< 0.001
Rural	3.60	3.55–3.64		
*Highest education level*				
No education	5.02	4.91–5.13	724.55	< 0.001
Primary	3.53	3.49–3.57		
Secondary	2.53	2.30–2.40		
Higher	1.34	1.79–2.06		
*Household income*				
Poor	3.41	3.34–3.47	105.15	< 0.001
Middle income	3.73	3.67–3.79		
Rich	3.05	2.99–3.12		
*Husband/partner’s level of education*				
No education	4.49	4.36–4.62	433. 84	< 0.001
Primary	3.82	3.77–3.87		
Secondary	2.72	2.67–2.77		
Higher	2.40	2.29–2.52		
*Employed*				
No	3.11	3.05–3.17	176.19	< 0.001
Yes	3.62	3.58–3.66		
*Exposure to mass media – radio listening*				
Not at all	3.58	3.53–3.64	26.01	< 0.001
Less than once a week	3.37	3.29–3.46		
At least once a week	3.30	3.24–3.36		
*Exposure to mass media – television watching*				
Not at all	3.57	3.53–3.61	101.42	< 0.001
Less than once a week	3.18	3.06–3.30		
At least once a week	2.82	2.74–2.900		

Statistically significant at P < 0.05, where SD is standard deviation and CI is confidence interval of the mean

The analysis also revealed that age at first cohabitation, modern contraceptive use, type of place of residence (rural or urban), respondent’s education level, household economic status, education level of the husband or partner, employment status and exposure to mass media (thus radio listening and watching television) were significantly associated with number of children ever born. However, religion and ethnicity were not significantly associated with fertility (number of children ever born). It should be noted that all control variables found insignificant were not included for multivariable Poisson regression modeling analysis.

### Determinants of fertility: Poisson regression model

Table 3 shows the results from Poisson regression models, which are incidence rate ratios of various explanatory variables for the expected number of children ever born among women aged 15–49 years who were married or cohabiting. Model 1, which is unadjusted model, indicates that women’s autonomy in household decision making was not associated with the number of children ever born. On the other hand, women’s sexual autonomy was significantly associated with the number of children ever born. Using women with no autonomy as a reference category, women with autonomy (UIRR = 0.69, CI 0.64–0.75) and women with partial autonomy (UIRR = 0.87, CI 0.79–0.96) were likely to have fewer number of children in their lifetime.

**Table 3:** The incidence rate ratio of women’s autonomy in the household and other explanatory variables predicting the likelihood of fertility: Poisson regression models

Variables	Fertility (Children ever born)					
	Unadjusted (Model 1)		Adjusted (model 2)		Adjusted (Model 3)	
	UIRR	95% C.I	AIRR	95% C.I	AIRR	95% C.I
*Autonomy in household decision making participation*						
Have autonomy	1.07	0.98–1.17	1.02	0.99–1.05	1.02	0.99–1.05
Have partial autonomy	1.04	0.95–1.13	1.01	0.98–1.03	1.01	0.99–1.04
Have no autonomy	1.00		1.00		1.00	
*Sexual autonomy*						
Have autonomy	0.69***	0.64–0.75	0.90***	0.88–0.92	0.98	0.95–1.00
Have partial autonomy	0.87***	0.79–0.96	0.96***	0.94–0.99	1.00	0.97–1.02
Have no autonomy	1.00		1.00		1.00	
*Age at first cohabitation/marriage*						
19 year and less			1.17***	1.14–1.19	1.15***	1.13–1.18
20 years and above			1.00		1.00	
*Contraceptive use*						
Using modern methods			1.19***	1.17–1.21	1.17***	1.15–1.19
Not using modern			1.00		1.00	
*Type of place of residence*						
Urban					0.91***	0.88–0.93
Rural					1.00	
*Highest education level*						
No education					1.83***	1.67–1.99
Primary					1.55***	1.42–1.69
Secondary					1.23***	1.13–1.33
Higher					1.00	
*Household economic status*						
Poor					1.05***	1.01–1.09
Middle income					1.00	0.99–1.07
Rich					1.00	
*Husband/partner’s level of education*						
No education					1.23***	1.16–1.30
Primary					1.18***	1.12–1.24
Secondary					1.02	0.97–1.07
Higher					1.00	
*Employed*						
No					0.95	0.91–1.00
Yes					1.00	
*Exposure to media-radio listening*						
Not at all					1.01	0.99–1.03
Less than once a week					1.00	0.98–1.03
At least once a week					1.00	
*Exposure to media-watching television*						
Not at all					1.04	0.99–1.08
Less than once a week					1.02	0.97–1.06
At least once a week					1.00	

***Statistically significant at P < 0.05

Model 2, which is adjusted for proximate determinants of fertility, thus contraceptive use and age at first marriage, indicates that women’s autonomy in household decision making was not associated with the number of children ever born. On the other hand, women’s sexual autonomy was significantly associated with the number of children ever born. Using women with no autonomy as a reference category, women with autonomy (AIRR = 0.90, CI 0.88–0.92) and women with partial autonomy (AIRR = 0.96, CI 0.94–0.99) were likely to have fewer number of children in their lifetime. Meanwhile, age at first cohabitation was significantly associated with the number of children ever born. Using women who started cohabiting at the age of 20 years and above as a reference category, women who started cohabiting at the age of 19 years or less (AIRR = 1.17, CI 1.14–1.19) were likely to have higher number of children in their lifetime. Furthermore, the use of modern methods of contraceptives was significantly associated with the number of children ever born. Using women who were not using modern methods of contraceptives as a reference category, women who were using modern methods of contraceptives (AIRR = 1.19, CI 1.17–1.21) were likely to have higher number of children.

The final Model 3 shows the net effects of all explanatory variables. The dimensions of women’s autonomy such as decision-making, and sexual autonomy were not determinants of number of children ever born. This seems to imply that background characteristics, especially education level of the woman or partner, rural–urban residence and household economic status (which are significant with model 3) may have been moderating factors for the sexual autonomy. On the other hand, like with model 2, proximate determinants of fertility (age at first cohabitation and use of modern contraceptives) were still significant with number of children ever born conforming to the conceptual framework.

Additionally, model 3 also shows that other explanatory factors such as rural–urban place of residence, education level of the woman or partner and household economic status were determinants of the number of children ever born. This also suggests that these variables are very important in moderating either woman’s autonomy in the household and fertility nexus. Thus, women who were residing in rural areas as a reference category, women who were living in urban areas (IRR = 0.91, CI 0.88–0.93) were likely to have fewer number of children in their life-time. On women’s education level, using higher education level as a reference category, women with no education (IRR = 1.83, CI 1.67–1.99) or primary education (IRR = 1.55, CI 1.42–1.69) or secondary education (IRR = 1.23, CI 1.13–1.33) were likely to have higher number of children. Moreover, women whose husbands or partners had no education (IRR = 1.13, CI 1.05–1.21) or primary education (IRR = 1.13, CI 1.06–1.20) were likely to have higher number of children compared to women whose partners had higher education (reference category). Nonetheless, there was no significant difference of number of children ever born between women whose partners had secondary education and women of reference category. With respect to household economic status, using women from rich households as a reference category, women from poor households (IRR = 1.05, CI 1.01–1.09) were likely to have higher number of children in their lifetime. However, there was no significant difference in the numbers of children ever born between women from middle income households and rich households.

The mediating variables thus, contraceptive use and age at first cohabitation, like with mode 2 are still significantly associated with model 3. Thus, women who started cohabiting at the age of 19 years or less (AIRR = 1.15, CI 1.13–1.18) and who were not using modern contraceptive methods (AIRR = 1.17, CI 1.15–1.19) were likely to have higher number of children. Meanwhile, some variables (employment status and exposure to mass media, thus listening to radio and watching television) were not determinants of the number of children ever born.

## Discussions

The main aim of this study was to identify the determinants of fertility. In particular, the paper examined if women’s autonomy in the household is the determinant of fertility. Thus, the data on married or cohabiting women at the time of survey was analyzed. Poisson’s regression models were used to predict if dimensions of women autonomy in the household (thus autonomy in household decision-making and sexual autonomy) are the determinants of the number of children ever born. The findings of the study show that about half of the respondents (49.1%) had full autonomy in household decision-making and slightly above one-third of the respondents (36.4%) had partial autonomy in household decision-making participation. The results are higher than other nations in Sub-Sahara Africa such as Guinea (33.7%), Zambia (36.3%) and Mali (10.6%) and lower than Namibia (68.4%) [107]. On the other hand, the findings also show that about two-third (64.0%) of the respondents had the sexual autonomy and about one-fifth (18.7%) of the respondents had partial sexual autonomy. The results, in relation to the proportion (82.7%) of women with either complete sexual autonomy or partial sexual autonomy in Malawi, are almost similar to other countries in Sub-Sahara Africa thus Zambia (79.8%) and Togo (84.3%); higher than other nations thus Mali (45.2%) and Burkina Faso (40.0%); and lower than other nations thus Lesotho (97.7%) and Namibia (99.0%) [65]. This suggests that cohabiting women aged 15–49 years in Malawi who reported to have either partial or full autonomy is relatively better than other countries.

Surprisingly, the study found that women’s autonomy in household decision-making was not the determinant of the number of children ever born in Malawi. As discussed earlier, women autonomy in general expected to have a negative relationship with fertility in most contexts. However, the findings in this study seem to indicate that the relationship between women autonomy and fertility seems to vary depending on sociocultural context. Though the level of autonomy in household decision is higher in Malawi, the study did not find an association with fertility. This seems to indicate that interaction of sociocultural environments determines the association between women autonomy and fertility in the context of Malawi. The finding is consistent with a study in Tanzania, which found no significant association between household decision-making and fertility [108]. But in another context in Zimbabwe, inverse association between women autonomy and fertility was found [25]. Nonetheless, women’s autonomy in the household is not achieved in vacuum, rather there are underlying factors that enhance women’s autonomy in the household. Evidenced by a study that found that social norms reinforced by patriarchy in India and developing countries inhibit women realization of autonomy in the household and beyond [109]. Thus, even in instances where women claim to have autonomy in all household decision-making may not be true in reality rather this may be coerced or consulted for an input thus, husbands or partners still make the final say or are the major decision makers.

Women’s sexual autonomy was found to be a determinant of the number of children ever born in the model 2, controlling the proximate determinants. This shows that women’s sexual autonomy seems to affect fertility through the proximate determinants as expected. However, women’s sexual autonomy was found not to be a determinant of the number of children ever born after adjusted for all covariates. The finding is consistent with studies in other context where it was found no significant association between number of children ever born and sexual autonomy in Tanzania [110]; in Uganda [111] and in Cote d’Ivoire and Nigeria [112]. Furthermore, the findings also affirm the findings in South-West Nigeria [113] and Zambia [114]. The authors have argued that women’s claim of sexual autonomy may not necessarily be true as they are more likely to succeed in refusing sex or ask for condom use through bargaining or begging with reasons like sickness, not in the mood, tiredness and menstrual pain [113, 114]. Nonetheless, such excuses may not be of common occurrences as they may raise suspicions of infidelity and lack of trust, which could lead to sexual violence within the marriage. Hence, it can be expected with lack of real autonomy, women’s claim of sexual autonomy may not have significant influence on number of children ever born as observed in Malawi. However, the study [115] observed that women who refuse sex have significantly fewer number of children ever born in Uganda and Kenya. However, the study has limitation since it only used women refusal of sex but did not include asking partner to use condom for measuring sexual autonomy.

The insignificant relationship between dimensions of women’s autonomy and children ever born in Malawi can be attributed to cultural norms, like patriarchy and religion, which advocates men as the heads of the households and women should be submissive [116]. For instance, despite mixed marriage systems among ethnic groups (patrilineal and matrilineal), both are grounded in patriarchy where in patrilineal the husband has absolute authority in the household and in matrilineal the uncle of the woman has higher authority. This may inhibit true autonomy of women in the households. The prevailing cultural norms in Malawi can be attributed to high proportion (84%) of national population residing in rural areas and not educated (78%) to secondary or higher level [117]. In rural areas, cultural practices are dominant, while women with less than secondary education level are less likely to question and challenge the infringing cultural norms. This is likely to deny women to have absolute autonomy in the household, thus contributing to their inability to negotiate and realize their desired reproductive health outcomes including low fertility. Meanwhile, women living in urban areas, with at least secondary education level and from middle and rich households were significantly associated with fewer number of children ever born.

Place of residence, as alluded to, has been found as a determinant of the number of children ever born. Women who lived in urban areas were significantly associated with having fewer number of children ever born than their counterparts who lived in rural areas. This is consistent with other studies done in Zimbabwe [25] and in Uganda and Kenya [115].

Women’s education level was found significant with the number of children ever born. This is consistent with other studies done in other sub-Sahara African countries that found increase in women’s education level, especially secondary or higher education level, is inversely and significantly associated with number of children ever born thus in Zimbabwe [25] in Uganda and Kenya [115] and in Nigeria [118]. Thus, the more the time women spend schooling, the more likely they are to get married late hence they start giving births late thus they have reduced fecundity. Additionally, when women are more educated, they tend to have formal employment with good wages or reward which commensurate with their active presence at work and this motivates them to forgo having children hence they are more likely to have fewer number of children ever born. Furthermore, the more educated women are, the more likely they are to be informed on issue of reproductive health resulting in fewer children ever born.

Women autonomy in the household could be one of the moderating factors for education level-low fertility nexus. Thus, having secondary or higher education level especially among women enhance them to have cognitive abilities which are essential to their capacity to participate, to reflect on and act on the conditions of their lives and gain access to knowledge, information and ideas that would help them to make informed decisions and also claim their autonomy [117]. For instance, in Bangladesh [119]; in Nigeria [120] and in Burkina Faso [121] found increase in women education especially secondary education level or higher as an enabling factor for women’s autonomy in household and fertility. Furthermore, women with secondary education level or higher question challenge cultural norms that infringes them, hence they claim their autonomy and are likely to achieve their desired fertility (which is often low) even if their partners wish otherwise. Therefore, it can be deduced that there is linkage among education level, women’s autonomy in the household and fertility thus, children ever born.

Household economic status was found as a determinant of the number of children ever born. Thus, women from rich and middle-income households were inversely and significantly associated with the number of children ever born. This finding is consistent with other studies done in other sub-Sahara African countries, thus in Kenya [122] and in Uganda and Kenya [106]. Moreover, the finding is also consistent with other study findings in Malawi [88].

Moreover, the association between household economic status and fertility thus, children ever born can be argued to be moderated by women’s autonomy in the household. Werwath (2011) argues that increase in economic status is highly correlated with exposure to mass media, as can afford to have radios television sets [49]. This helps women to listen to behavioral changing information hence are likely to be more informed on topical issues like reproductive health and human rights among others, thus are relatively freed from the burdening tradition norms. Hence, women can relatively have autonomy in the household than women from poor households. Thus, with the autonomy women tend to be very rational about decision-making including fertility decisions, thus they are likely to opt for fewer number of children.

Therefore, it can be deduced and concluded that residing in urban areas, at least secondary education for women and from middle or rich household moderate women’s autonomy in the household which in turn affect fertility thus, children ever born. Meanwhile, other variables, thus employment status and exposure to mass media (radio listening and watching television) were not determinants of the number of children ever born.

### Strengths and limitations of the study

The strength of the study is derived from its use of DHS dataset which collects data based on well calculated and standardized sample, hence the generalization of the findings is more reliable. Moreover, the use of Poisson regression model, suited for outcome variable with count data, gives reliable results with respect to the nexus of explanatory variables and number of children ever born. Nonetheless, there are some limitations associated with the study. First, the data was obtained based on self-reported, which may have a bearing on the results. This is because there was the possibility of social desirability inherent with self-reporting from the respondents. However, the DHS has taken due care to collect accurate information and evaluated the quality of the reporting of the information by the respondents. Another weakness is that women’s autonomy is a complex phenomenon even in household context, as such there is no consensus on definition or the most important dimensions of autonomy. Thus, women autonomy may not have independent effect on children ever born as it may be interacting with sociocultural norms prevailing in the society. Since we do not have a robust measurable indicator for reflecting sociocultural norms prevailing in the society, we have not tested for interaction effect.

## Conclusions

The level of women autonomy is greater is Malawi in terms of household decision-making and sexual autonomy. However, women’s autonomy is not associated with the fertility in the context of Malawi. However, the study has argued that having at least secondary education or higher, living in urban and middle or rich households could be moderate women’s autonomy in household especially sexual autonomy on fertility. Moreover, these stated moderating variables are also directly associated with fertility. On the other hand, age at first cohabitation and use of contraceptives mediates the association. The higher level of women autonomy and its insignificant association with fertility seems to indicate that the cultural factors may be preventing the women to make a reproductive choice and therefore lack reproductive rights.

The study recommends the Government of Malawi should come up with economic hardship emancipation policy for poor households. The government should also come up with a girl-child secondary school completion policy. Furthermore, the government should accelerate the implementation, monitoring and evaluation of National Gender Policy to ensure the women empowerment/autonomy is having positive effect at all levels including the community and household levels thus, eradicating the women-burdening cultural values. The government should also foster the continued use of the modern contraceptive use.

## Data Availability

The dataset used was requested from MEASURE DHS. https://www.dhsprogram.com/data/dataset_admin/login_main.cfm.

## Abbreviations

AIC: Akaike’s Information Criterion
ANOVA: Analyses of Variances
BIC: Bayesian Information Criterion
CEB: Children Ever Born
DHS: Demographic and Health Survey
EAs: Enumeration Areas
IRR: Incidence Rate Ratio
MDHS: Malawi Demographic and Health Survey
WCI: Wald Confidence Interval
